# Na_2.9_KMo_12_S_14_: a novel quaternary reduced molybdenum sulfide containing Mo_12_ clusters with a channel structure

**DOI:** 10.1107/S1600536813013603

**Published:** 2013-05-25

**Authors:** Patrick Gougeon, Philippe Gall, Diala Salloum

**Affiliations:** a Unité Sciences Chimiques de Rennes, UMR CNRS No. 6226, Université de Rennes I–INSA Rennes, Campus de Beaulieu, 35042 Rennes CEDEX, France; b Faculty of Science III, Lebanese University, PO Box 826, Kobbeh–Tripoli, Lebanon

## Abstract

The crystal structure of tris­odium potassium dodeca­molybdenum tetra­deca­sulfide, Na_2.9 (2)_KMo_12_S_14_, consists of Mo_12_S_14_S_6_ cluster units inter­connected through inter­unit Mo—S bonds and delimiting channels in which the Na^+^ cations are disordered. The cluster units are centered at Wyckoff positions 2*d* and have point-group symmetry 3.2. The K atom lies on sites with 3.2 symmetry (Wyckoff site 2*c*) between two consecutive Mo_12_S_14_S_6_ units. One of the three independent S atoms and one Na atom lie on sites with 3.. symmetry (Wyckoff sites 4*e* and 4*f*). The other Na atom occupies a 2*b* position with -3.. symmetry. The crystal studied was a merohedral twin with refined components of 0.4951 (13) and 0.5049 (13).

## Related literature

For a previous report on the compounds K_1+*x*_Mo_12_S_14_ (*x* = 0, 1.1, 1.3, and 1.6), see: Picard *et al.* (2006 ▶). For details of the *i*- and *a*-type ligand notation, see: Schäfer & von Schnering (1964 ▶). For the program *JANA2000*, see: Petříček & Dušek (2000 ▶). The twinning was identified using the TwinRotMat routine in *PLATON* (Spek, 2009 ▶).

## Experimental

### 

#### Crystal data

Na_2.90_KMo_12_S_14_
*M* *_r_* = 1705.89
Trigonal, 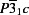

*a* = 9.3664 (1) Å
*c* = 16.2981 (2) Å
*V* = 1238.26 (2) Å^3^
*Z* = 2
Mo *K*α radiation
μ = 7.24 mm^−1^
*T* = 100 K
0.08 × 0.07 × 0.07 mm

#### Data collection

Nonius KappaCCD diffractometer
Absorption correction: analytical (de Meulenaer & Tompa, 1965 ▶) *T*_min_ = 0.550, *T*_max_ = 0.572
37291 measured reflections
2536 independent reflections
2376 reflections with *I* > 2σ(*I*)
*R*_int_ = 0.056

#### Refinement

*R*[*F* ^2^ > 2σ(*F* ^2^)] = 0.027
*wR*(*F* ^2^) = 0.073
*S* = 1.13
2536 reflections
52 parameters
Δρ_max_ = 2.74 e Å^−3^
Δρ_min_ = −1.84 e Å^−3^

### 

Data collection: *COLLECT* (Nonius, 1998 ▶); cell refinement: *COLLECT*; data reduction: *EVALCCD* (Duisenberg, 1998 ▶); program(s) used to solve structure: *SHELXS97* (Sheldrick, 2008 ▶); program(s) used to refine structure: *SHELXL97* (Sheldrick, 2008 ▶); molecular graphics: *DIAMOND* (Bergerhoff, 1996 ▶); software used to prepare material for publication: *SHELXL97*.

## Supplementary Material

Crystal structure: contains datablock(s) I, global. DOI: 10.1107/S1600536813013603/ru2051sup1.cif

Structure factors: contains datablock(s) I. DOI: 10.1107/S1600536813013603/ru2051Isup2.hkl

Additional supplementary materials:  crystallographic information; 3D view; checkCIF report

## Figures and Tables

**Table 1:** Selected bond lengths (Å)

Mo1—S3	2.3855 (10)
Mo1—S1^i^	2.4620 (8)
Mo1—S1	2.4822 (8)
Mo1—S1^ii^	2.4944 (8)
Mo1—S2	2.5907 (7)
Mo1—Mo1^iii^	2.6296 (5)
Mo1—Mo2	2.7155 (4)
Mo1—Mo2^i^	2.7803 (4)
Mo2—S1	2.4589 (7)
Mo2—S2	2.4655 (7)
Mo2—S2^iii^	2.4904 (8)
Mo2—S2^iv^	2.5866 (7)
Mo2—Mo2^v^	2.6440 (5)
Mo2—Mo2^iv^	2.6745 (5)
Mo2—Mo2^i^	2.6765 (4)
K1—S3	2.9460 (13)
K1—S2^vi^	3.4188 (7)
K1—S2^vii^	3.4188 (7)
K1—S2^viii^	3.4188 (7)
Na1—S2^ix^	3.3131 (17)
Na1—S1^x^	3.856 (12)
Na1—S2^xi^	3.898 (11)
Na2—S1^xii^	3.210 (12)
Na2—S2^xiii^	3.56 (2)

Symmetry codes: (i) 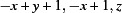; (ii) 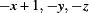; (iii) 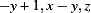; (iv) 
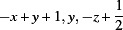
; (v) 
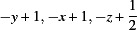
; (vi) 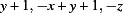; (vii) 
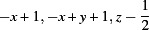
; (viii) 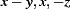; (ix) 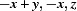; (x) 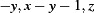; (xi) 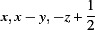; (xii) 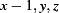; (xiii) 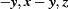.

**Fig. 1.**
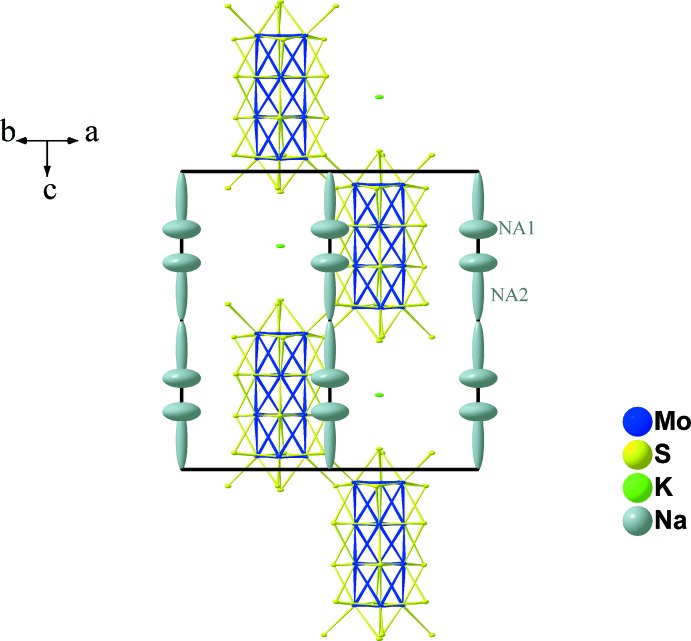
: View of Na2.9KMo12S14. Displacement ellipsoids are drawn at the 50% probability level.

**Fig. 2.**
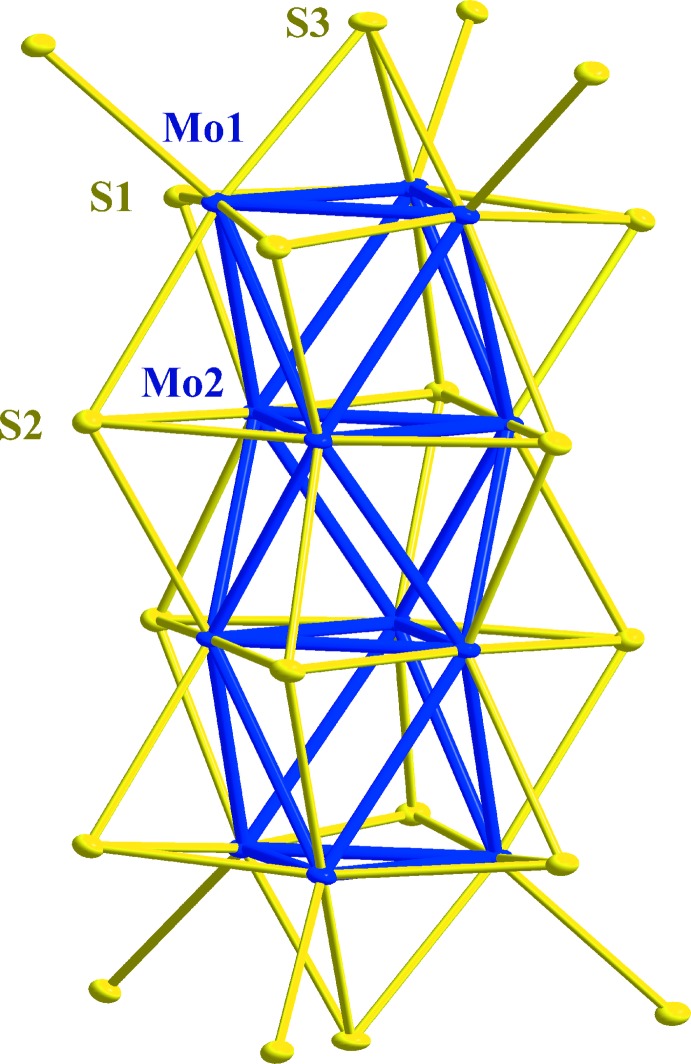
: Plot showing the atom-numbering scheme of the Mo12S14S6 cluster units. Displacement ellipsoids are drawn at the 50% probability level.

**Fig. 3.**
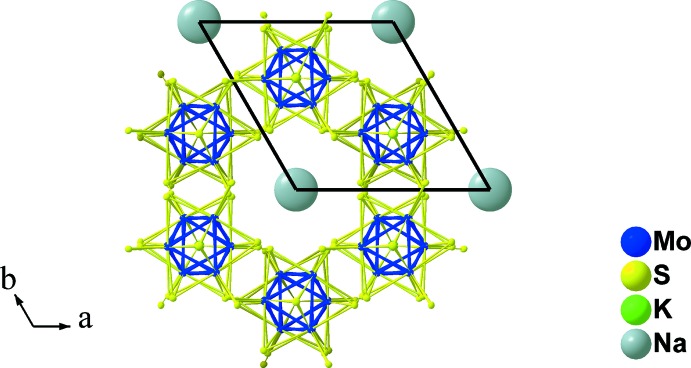
: View of Na2.9KMo12S14 along the c axis showing the channels. Displacement ellipsoids are drawn at the 50% probability level.

## supplementary crystallographic information

### Comment

In a previous paper, we reported the synthesis, the crystal structures and, the physical properties of the compounds K_1 +_*_x_*Mo_12_S_14_ (*x*= 0, 1.1, 1.3, and 1.6) which crystallize in a new structural type only based on the Mo_12_ cluster (Picard *et al.*, 2006). We present here the crystal structure of the sulfide Na_2.9_KMo_12_S_14_ which is isomorphous with the latter compounds (Picard *et al.*, 2006). Its crystal structure (Fig. 1) contains Mo_12_S^i^_14_S*^a^*_6_ cluster units (for details of the i- and a-type ligand notation, see Schäfer & von Schnering (1964)). The i-type ligands cap Mo triangular faces and the a-type ones are in apical position for the external Mo1 atoms (Fig. 2). The Mo_12_S_14_ cluster unit is centred at a 2 d (D_3_ or 32 symmetry) position. The Mo—Mo distances within the Mo_12_ clusters are 2.6296 (5) Å for the distances in the triangles formed by the Mo1 related through the threefold axis and 2.6764 (4) in the triangles formed by the Mo2 atoms. The distances between the triangles formed by the Mo1 and Mo2 atoms are 2.7155 (4) and 2.7803 (4) Å and those between the two Mo2_3_ triangles, 2.6440 (5) and 2.6745 (5) Å. The sulfur atoms bridge either one [S1 and S3] or two [S2] Mo triangular faces of the clusters. Moreover the S1 atoms are linked to a Mo atom of a neighboring cluster. The Mo—S bond distances range from 2.3855 (10) to 2.5907 (7) Å. Each Mo_12_S_14_ unit is interconnected to 6 adjacent ones *via* Mo1—S1 bonds to form the three-dimensional Mo—S framework, the connective formula of which is Mo_12_S^i^_8_S^i-a^_6/2_S^a-i^_6/2_. It results from this arrangement that the shortest intercluster Mo1—Mo1 distance between the Mo_12_ clusters is 3.4025 (3) Å, indicating only weak metal-metal interaction. The Na cations reside in large channels extending along the *c* axis (Fig. 3). The Na1 cations occupied distorted tri-capped trigonal prismatic cavities of sulfur atoms and the Na2 are in an octahedron compressed along the threefold axis. The Na—S distances spread over a wide range 3.210 (12) - 3.898 (11) Å. The K cation is eight-coordinated with six S2 atoms at 3.4188 (7), forming an octahedron compressed along the threefold axis, and the remaining two S3 atoms capping two opposite faces of the octahedron at 2.9460 (13).

### Experimental

Single crystals of Na_2.9_KMo_12_S_14_ were obtained by treating crystals of KMo_12_S_14_ in a basic reducing solution of Na_2_S_2_O_3_/NaOH at 333 K for 3 days. The KMo_12_S_14_ compound was prepared by oxidation of single crystals of K_2.3_Mo_12_S_14_ in an aqueous solution of iodine at 363 K for 48 h. Single crystals of K_2.3_Mo_12_S_14_ were prepared from a mixture of K_2_MoS_4_, MoS_2_, and Mo with the nominal composition K_2_Mo_3_S_4_. The initial mixture (*ca* 5 g) was cold pressed and loaded into a molybdenum crucible, which was sealed under a low argon pressure using an arc welding system. The charge was heated at the rate of 300 K/h up to 1773 K, temperature which was held for 6 h, then cooled at 100 K/h down to 1373 K and finally furnace cooled. All handlings of materials were done in an argon-filled glove box.

### Refinement

In the first stage of the refinement, the atomic positions of the Mo and S atoms were deduced from those in KMo_12_S_14_ (Picard *et al.*, 2006). A subsequent difference-Fourier synthesis reveals the potassium atom and a quasi-continuous electron density along the *c* axis due to the sodium atoms. The latter was modelled with two partly occupied sodium sites (4 e and 2 b positions) using second-order tensors for the anisotropic displacement parameters. Anharmonic treatment of the Na1 and Na2 atoms using the program JANA2000 (Petříček & Dušek, 2000) was unsuccessful. The final occupation factors for the Na atoms were refined freely. The highest peak and the deepest hole in the final Fourier map are located 1.07 Å from Na2 and 0.58 Å from Mo2, respectively. Analysis of the intensity data using the TwinRotMat routine of *PLATON* (Spek, 2009) revealed the studied crystal was twinned by merohedry with [100, 010, 001]as the twin matrix. The ratio of the twin components was refined to 0.4951 (13):0.5049 (13). The Na content found seems reliable since the cationic electron transfer towards the Mo_12_ cluster deduced from our refinement is +3.9 and is in agreement with the maximal limit of +4 that the Mo_12_ cluster can accept to be well bonded and with the semi-conductor behavior observed on a single-crystal. Indeed, a lower stoichiometry in Na would lead to a metallic behavior. This is also confirmed by semi-quantitative analyses by energy dispersive spectroscopy (eds) which indicated roughly stoichiometries comprised between 2.6 and 3.2 for the Na content.

### Crystal data

**Table:**

Na_2.90_KMo_12_S_14_	*D*_x_ = 4.575 Mg m^−^^3^
*M**_r_* = 1705.89	Mo *K*α radiation, λ = 0.71069 Å
Trigonal, *P*3_1_*c*	Cell parameters from 5780 reflections
*a* = 9.3664 (1) Å	θ = 3.5–39.8°
*c* = 16.2981 (2) Å	µ = 7.24 mm^−^^1^
*V* = 1238.26 (2) Å^3^	*T* = 100 K
*Z* = 2	Multi-faceted crystal, black
*F*(000) = 1558	0.08 × 0.07 × 0.07 mm

### Data collection

**Table:**

Nonius KappaCCD diffractometer	2536 independent reflections
Radiation source: fine-focus sealed tube	2376 reflections with *I* > 2σ(*I*)
Graphite monochromator	*R*_int_ = 0.056
φ scans (κ = 0) + additional ω scans	θ_max_ = 39.8°, θ_min_ = 3.5°
Absorption correction: analytical (de Meulenaer & Tompa, 1965)	*h* = −16→16
*T*_min_ = 0.550, *T*_max_ = 0.572	*k* = −16→16
37291 measured reflections	*l* = −29→27

### Refinement

**Table:**

Refinement on *F*^2^	Primary atom site location: structure-invariant direct methods
Least-squares matrix: full	Secondary atom site location: difference Fourier map
*R*[*F*^2^ > 2σ(*F*^2^)] = 0.027	*w* = 1/[σ^2^(*F*_o_^2^) + (0.029*P*)^2^ + 4.9317*P*] where *P* = (*F*_o_^2^ + 2*F*_c_^2^)/3
*wR*(*F*^2^) = 0.073	(Δ/σ)_max_ = 0.001
*S* = 1.13	Δρ_max_ = 2.74 e Å^−^^3^
2536 reflections	Δρ_min_ = −1.84 e Å^−^^3^
52 parameters	Extinction correction: *SHELXL97* (Sheldrick, 2008), Fc^*^=kFc[1+0.001xFc^2^λ^3^/sin(2θ)]^-1/4^
0 restraints	Extinction coefficient: 0.00032 (9)

### Special details

**Table:**

Geometry. All e.s.d.'s (except the e.s.d. in the dihedral angle between two l.s. planes) are estimated using the full covariance matrix. The cell e.s.d.'s are taken into account individually in the estimation of e.s.d.'s in distances, angles and torsion angles; correlations between e.s.d.'s in cell parameters are only used when they are defined by crystal symmetry. An approximate (isotropic) treatment of cell e.s.d.'s is used for estimating e.s.d.'s involving l.s. planes.
Refinement. Refinement of *F*^2^ against ALL reflections. The weighted *R*-factor *wR* and goodness of fit *S* are based on *F*^2^, conventional *R*-factors *R* are based on *F*, with *F* set to zero for negative *F*^2^. The threshold expression of *F*^2^ > σ(*F*^2^) is used only for calculating *R*-factors(gt) *etc*. and is not relevant to the choice of reflections for refinement. *R*-factors based on *F*^2^ are statistically about twice as large as those based on *F*, and *R*- factors based on ALL data will be even larger.

### Fractional atomic coordinates and isotropic or equivalent isotropic displacement parameters (Å^2^)

**Table:**

	*x*	*y*	*z*	*U*_iso_*/*U*_eq_	Occ. (<1)
Mo1	0.50983 (3)	0.16646 (3)	0.043656 (15)	0.00973 (6)	
Mo2	0.66853 (3)	0.16929 (3)	0.183610 (15)	0.00826 (5)	
S1	0.68780 (10)	0.04088 (9)	0.05564 (4)	0.01069 (11)	
S2	0.36482 (9)	0.02520 (9)	0.17966 (4)	0.01064 (11)	
S3	0.6667	0.3333	−0.06924 (8)	0.0129 (2)	
K1	0.6667	0.3333	−0.2500	0.0195 (3)	
Na1	0.0000	0.0000	0.1936 (13)	0.36 (4)	0.71 (5)
Na2	0.0000	0.0000	0.099 (3)	0.40 (4)	0.74 (4)

### Atomic displacement parameters (Å^2^)

**Table:**

	*U*^11^	*U*^22^	*U*^33^	*U*^12^	*U*^13^	*U*^23^
Mo1	0.01224 (10)	0.01354 (11)	0.00434 (9)	0.00714 (8)	−0.00045 (7)	0.00041 (7)
Mo2	0.01081 (9)	0.01033 (10)	0.00371 (9)	0.00535 (8)	−0.00034 (6)	−0.00060 (7)
S1	0.0143 (3)	0.0125 (3)	0.0063 (2)	0.0074 (2)	−0.0003 (2)	−0.00134 (19)
S2	0.0121 (2)	0.0118 (2)	0.0064 (2)	0.0047 (2)	−0.0014 (2)	−0.0007 (2)
S3	0.0172 (3)	0.0172 (3)	0.0044 (4)	0.00859 (16)	0.000	0.000
K1	0.0264 (5)	0.0264 (5)	0.0058 (6)	0.0132 (3)	0.000	0.000
Na1	0.47 (6)	0.47 (6)	0.12 (2)	0.24 (3)	0.000	0.000
Na2	0.045 (4)	0.045 (4)	1.12 (12)	0.023 (2)	0.000	0.000

### Geometric parameters (Å, º)

**Table:**

Mo1—S3	2.3855 (10)	S3—Mo1^iii^	2.3855 (10)
Mo1—S1^i^	2.4620 (8)	S3—Mo1^i^	2.3855 (10)
Mo1—S1	2.4822 (8)	K1—S3	2.9460 (13)
Mo1—S1^ii^	2.4944 (8)	K1—S3^vii^	2.9460 (13)
Mo1—S2	2.5907 (7)	K1—S2^viii^	3.4188 (7)
Mo1—Mo1^iii^	2.6296 (5)	K1—S2^ix^	3.4188 (7)
Mo1—Mo1^i^	2.6296 (5)	K1—S2^x^	3.4188 (7)
Mo1—Mo2	2.7155 (4)	K1—S2^ii^	3.4188 (7)
Mo1—Mo2^i^	2.7803 (4)	K1—S2^xi^	3.4188 (7)
Mo2—S1	2.4589 (7)	K1—S2^xii^	3.4188 (7)
Mo2—S2	2.4655 (7)	Na1—Na2	1.55 (5)
Mo2—S2^iii^	2.4904 (8)	Na1—Na1^xiii^	1.84 (4)
Mo2—S2^iv^	2.5866 (7)	Na1—S2^xiv^	3.3131 (17)
Mo2—Mo2^v^	2.6440 (5)	Na1—S2^xv^	3.3131 (17)
Mo2—Mo2^iv^	2.6745 (5)	Na1—S1^xvi^	3.856 (12)
Mo2—Mo2^iii^	2.6765 (4)	Na1—S1^xvii^	3.856 (12)
Mo2—Mo2^i^	2.6765 (4)	Na1—S1^i^	3.856 (12)
Mo2—Mo1^iii^	2.7803 (4)	Na1—S2^xviii^	3.898 (11)
S1—Mo1^iii^	2.4620 (8)	Na1—S2^xiii^	3.898 (11)
S1—Mo1^ii^	2.4944 (8)	Na1—S2^xix^	3.898 (11)
S1—Na2^vi^	3.210 (12)	Na2—S1^i^	3.210 (12)
S2—Mo2^i^	2.4904 (8)	Na2—S1^xvi^	3.210 (12)
S2—Mo2^iv^	2.5866 (7)	Na2—S1^xvii^	3.210 (12)
S2—Na1	3.3131 (17)	Na2—S2^xiv^	3.56 (2)
S2—K1^ii^	3.4188 (7)	Na2—S2^xv^	3.56 (2)
			
S3—Mo1—S1^i^	92.320 (19)	Mo1^iii^—S1—Mo1^ii^	127.81 (3)
S3—Mo1—S1	91.819 (19)	Mo1—S1—Mo1^ii^	84.55 (3)
S1^i^—Mo1—S1	169.89 (3)	Mo2—S1—Na2^vi^	99.6 (9)
S3—Mo1—S1^ii^	89.07 (3)	Mo1^iii^—S1—Na2^vi^	98.08 (6)
S1^i^—Mo1—S1^ii^	93.84 (4)	Mo1—S1—Na2^vi^	160.3 (4)
S1—Mo1—S1^ii^	95.45 (3)	Mo1^ii^—S1—Na2^vi^	114.5 (6)
S3—Mo1—S2	171.28 (3)	Mo2—S2—Mo2^i^	65.37 (2)
S1^i^—Mo1—S2	84.81 (3)	Mo2—S2—Mo2^iv^	63.873 (19)
S1—Mo1—S2	89.74 (2)	Mo2^i^—S2—Mo2^iv^	62.735 (18)
S1^ii^—Mo1—S2	99.32 (2)	Mo2—S2—Mo1	64.912 (19)
S3—Mo1—Mo1^iii^	56.554 (16)	Mo2^i^—S2—Mo1	66.315 (19)
S1^i^—Mo1—Mo1^iii^	118.03 (2)	Mo2^iv^—S2—Mo1	118.36 (3)
S1—Mo1—Mo1^iii^	57.50 (2)	Mo2—S2—Na1	154.66 (8)
S1^ii^—Mo1—Mo1^iii^	131.58 (2)	Mo2^i^—S2—Na1	89.74 (2)
S2—Mo1—Mo1^iii^	117.822 (17)	Mo2^iv^—S2—Na1	101.5 (3)
S3—Mo1—Mo1^i^	56.554 (16)	Mo1—S2—Na1	110.7 (3)
S1^i^—Mo1—Mo1^i^	58.24 (2)	Mo2—S2—K1^ii^	92.19 (2)
S1—Mo1—Mo1^i^	117.29 (2)	Mo2^i^—S2—K1^ii^	150.01 (3)
S1^ii^—Mo1—Mo1^i^	130.870 (19)	Mo2^iv^—S2—K1^ii^	90.10 (2)
S2—Mo1—Mo1^i^	115.293 (18)	Mo1—S2—K1^ii^	123.66 (3)
Mo1^iii^—Mo1—Mo1^i^	60.0	Na1—S2—K1^ii^	109.13 (14)
S3—Mo1—Mo2	119.13 (2)	Mo1^iii^—S3—Mo1	66.89 (3)
S1^i^—Mo1—Mo2	113.78 (2)	Mo1^iii^—S3—Mo1^i^	66.89 (3)
S1—Mo1—Mo2	56.254 (17)	Mo1—S3—Mo1^i^	66.89 (3)
S1^ii^—Mo1—Mo2	137.91 (2)	Mo1^iii^—S3—K1	140.47 (2)
S2—Mo1—Mo2	55.315 (17)	Mo1—S3—K1	140.47 (2)
Mo1^iii^—Mo1—Mo2	62.662 (9)	Mo1^i^—S3—K1	140.47 (2)
Mo1^i^—Mo1—Mo2	91.183 (8)	S3—K1—S3^vii^	180.0
S3—Mo1—Mo2^i^	116.65 (2)	S3—K1—S2^viii^	70.407 (11)
S1^i^—Mo1—Mo2^i^	55.546 (18)	S3^vii^—K1—S2^viii^	109.593 (11)
S1—Mo1—Mo2^i^	114.41 (2)	S3—K1—S2^ix^	109.593 (11)
S1^ii^—Mo1—Mo2^i^	138.42 (2)	S3^vii^—K1—S2^ix^	70.407 (11)
S2—Mo1—Mo2^i^	55.112 (17)	S2^viii^—K1—S2^ix^	171.43 (2)
Mo1^iii^—Mo1—Mo2^i^	89.759 (7)	S3—K1—S2^x^	109.593 (11)
Mo1^i^—Mo1—Mo2^i^	60.181 (8)	S3^vii^—K1—S2^x^	70.407 (11)
Mo2—Mo1—Mo2^i^	58.275 (11)	S2^viii^—K1—S2^x^	63.43 (2)
S1—Mo2—S2	93.26 (3)	S2^ix^—K1—S2^x^	109.349 (11)
S1—Mo2—S2^iii^	87.06 (3)	S3—K1—S2^ii^	70.407 (11)
S2—Mo2—S2^iii^	173.83 (2)	S3^vii^—K1—S2^ii^	109.593 (11)
S1—Mo2—S2^iv^	117.76 (2)	S2^viii^—K1—S2^ii^	109.349 (11)
S2—Mo2—S2^iv^	90.68 (3)	S2^ix^—K1—S2^ii^	63.43 (2)
S2^iii^—Mo2—S2^iv^	94.62 (3)	S2^x^—K1—S2^ii^	78.22 (2)
S1—Mo2—Mo2^v^	144.46 (2)	S3—K1—S2^xi^	109.593 (11)
S2—Mo2—Mo2^v^	120.668 (19)	S3^vii^—K1—S2^xi^	70.407 (11)
S2^iii^—Mo2—Mo2^v^	60.414 (17)	S2^viii^—K1—S2^xi^	78.22 (2)
S2^iv^—Mo2—Mo2^v^	56.852 (18)	S2^ix^—K1—S2^xi^	109.349 (11)
S1—Mo2—Mo2^iv^	150.83 (2)	S2^x^—K1—S2^xi^	109.349 (12)
S2—Mo2—Mo2^iv^	60.265 (17)	S2^ii^—K1—S2^xi^	171.43 (2)
S2^iii^—Mo2—Mo2^iv^	120.634 (19)	S3—K1—S2^xii^	70.407 (11)
S2^iv^—Mo2—Mo2^iv^	55.862 (18)	S3^vii^—K1—S2^xii^	109.593 (11)
Mo2^v^—Mo2—Mo2^iv^	60.428 (12)	S2^viii^—K1—S2^xii^	109.349 (11)
S1—Mo2—Mo2^iii^	115.260 (19)	S2^ix^—K1—S2^xii^	78.22 (2)
S2—Mo2—Mo2^iii^	117.738 (19)	S2^x^—K1—S2^xii^	171.43 (2)
S2^iii^—Mo2—Mo2^iii^	56.865 (19)	S2^ii^—K1—S2^xii^	109.349 (11)
S2^iv^—Mo2—Mo2^iii^	117.009 (17)	S2^xi^—K1—S2^xii^	63.43 (2)
Mo2^v^—Mo2—Mo2^iii^	60.350 (10)	Na2—Na1—Na1^xiii^	180.000 (2)
Mo2^iv^—Mo2—Mo2^iii^	89.670 (5)	Na2—Na1—S2^xiv^	86.1 (4)
S1—Mo2—Mo2^i^	119.02 (2)	Na1^xiii^—Na1—S2^xiv^	93.9 (4)
S2—Mo2—Mo2^i^	57.760 (19)	Na2—Na1—S2	86.1 (4)
S2^iii^—Mo2—Mo2^i^	116.843 (19)	Na1^xiii^—Na1—S2	93.9 (4)
S2^iv^—Mo2—Mo2^i^	115.061 (17)	S2^xiv^—Na1—S2	119.54 (9)
Mo2^v^—Mo2—Mo2^i^	90.323 (5)	Na2—Na1—S2^xv^	86.1 (4)
Mo2^iv^—Mo2—Mo2^i^	59.222 (10)	Na1^xiii^—Na1—S2^xv^	93.9 (4)
Mo2^iii^—Mo2—Mo2^i^	60.0	S2^xiv^—Na1—S2^xv^	119.54 (9)
S1—Mo2—Mo1	57.07 (2)	S2—Na1—S2^xv^	119.54 (9)
S2—Mo2—Mo1	59.773 (17)	Na2—Na1—Na2^xiii^	180.000 (2)
S2^iii^—Mo2—Mo1	115.73 (2)	Na1^xiii^—Na1—Na2^xiii^	0.000 (2)
S2^iv^—Mo2—Mo1	147.71 (2)	S2^xiv^—Na1—Na2^xiii^	93.9 (4)
Mo2^v^—Mo2—Mo1	147.951 (10)	S2—Na1—Na2^xiii^	93.9 (4)
Mo2^iv^—Mo2—Mo1	111.157 (13)	S2^xv^—Na1—Na2^xiii^	93.9 (4)
Mo2^iii^—Mo2—Mo1	90.180 (8)	Na1—Na2—S1^i^	102.6 (9)
Mo2^i^—Mo2—Mo1	62.075 (8)	Na1—Na2—S1^xvii^	102.6 (9)
S1—Mo2—Mo1^iii^	55.650 (19)	S1^i^—Na2—S1^xvii^	115.4 (7)
S2—Mo2—Mo1^iii^	116.776 (19)	Na1—Na2—S1^xvi^	102.6 (9)
S2^iii^—Mo2—Mo1^iii^	58.573 (17)	S1^i^—Na2—S1^xvi^	115.4 (7)
S2^iv^—Mo2—Mo1^iii^	151.11 (2)	S1^xvii^—Na2—S1^xvi^	115.4 (7)
Mo2^v^—Mo2—Mo1^iii^	110.082 (13)	Na1—Na2—Na2^xx^	180.000 (2)
Mo2^iv^—Mo2—Mo1^iii^	144.903 (10)	S1^i^—Na2—Na2^xx^	77.4 (9)
Mo2^iii^—Mo2—Mo1^iii^	59.650 (8)	S1^xvii^—Na2—Na2^xx^	77.4 (9)
Mo2^i^—Mo2—Mo1^iii^	88.804 (7)	S1^xvi^—Na2—Na2^xx^	77.4 (9)
Mo1—Mo2—Mo1^iii^	57.157 (12)	Na1—Na2—Na1^xiii^	0.000 (1)
Mo2—S1—Mo1^iii^	68.80 (2)	S1^i^—Na2—Na1^xiii^	102.6 (9)
Mo2—S1—Mo1	66.67 (2)	S1^xvii^—Na2—Na1^xiii^	102.6 (9)
Mo1^iii^—S1—Mo1	64.26 (2)	S1^xvi^—Na2—Na1^xiii^	102.6 (9)
Mo2—S1—Mo1^ii^	136.36 (4)	Na2^xx^—Na2—Na1^xiii^	180.000 (1)

Symmetry codes: (i) −*x*+*y*+1, −*x*+1, *z*; (ii) −*x*+1, −*y*, −*z*; (iii) −*y*+1, *x*−*y*, *z*; (iv) −*x*+*y*+1, *y*, −*z*+1/2; (v) −*y*+1, −*x*+1, −*z*+1/2; (vi) *x*+1, *y*, *z*; (vii) −*y*+1, −*x*+1, −*z*−1/2; (viii) *y*+1, −*x*+*y*+1, −*z*; (ix) *x*−*y*, −*y*, *z*−1/2; (x) *y*+1, *x*, *z*−1/2; (xi) −*x*+1, −*x*+*y*+1, *z*−1/2; (xii) *x*−*y*, *x*, −*z*; (xiii) −*y*, −*x*, −*z*+1/2; (xiv) −*x*+*y*, −*x*, *z*; (xv) −*y*, *x*−*y*, *z*; (xvi) −*y*, *x*−*y*−1, *z*; (xvii) *x*−1, *y*, *z*; (xviii) *x*, *x*−*y*, −*z*+1/2; (xix) −*x*+*y*, *y*, −*z*+1/2; (xx) −*x*, −*y*, −*z*.
